# Acute Kidney Injury Definition and Diagnosis: A Narrative Review

**DOI:** 10.3390/jcm7100307

**Published:** 2018-09-28

**Authors:** Joana Gameiro, Jose Agapito Fonseca, Sofia Jorge, Jose Antonio Lopes

**Affiliations:** Division of Nephrology and Renal Transplantation, Department of Medicine Centro Hospitalar Lisboa Norte, EPE, Av. Prof. Egas Moniz, 1649-035 Lisboa, Portugal; jose.nuno.agapito@gmail.com (J.A.F.); sofiacjorge@sapo.pt (S.J.); jalopes93@hotmail.com (J.A.L.)

**Keywords:** acute kidney injury, definition, incidence, classification

## Abstract

Acute kidney injury (AKI) is a complex syndrome characterized by a decrease in renal function and associated with numerous etiologies and pathophysiological mechanisms. It is a common diagnosis in hospitalized patients, with increasing incidence in recent decades, and associated with poorer short- and long-term outcomes and increased health care costs. Considering its impact on patient prognosis, research has focused on methods to assess patients at risk of developing AKI and diagnose subclinical AKI, as well as prevention and treatment strategies, for which an understanding of the epidemiology of AKI is crucial. In this review, we discuss the evolving definition and classification of AKI, and novel diagnostic methods.

## 1. Introduction

Acute kidney injury (AKI) is a complex syndrome characterized by a decrease in renal function, associated with numerous etiologies and pathophysiological mechanisms [1,2]. It is a common diagnosis in hospitalized patients, associated with poorer short- and long-term outcomes and increased health care costs [3].

The incidence of AKI has increased in recent years [2,3]. However, there is significant variability in the reported incidence of AKI, which is associated with the different characteristics of the populations studied, cause of AKI, and diagnostic criteria used [1,2,3,4]. Additionally, the lack of studies assessing AKI in community settings and comparing critically ill and non-critical patients hampers the characterization of the epidemiology of AKI [2,3,4].

The importance of recognizing AKI applies to pediatric and adult patients, as well as ambulatory, hospitalized, and critically ill patients in multiple clinical settings, due to its prognostic impact [4,5,6]. The incidence of AKI is lowest in ambulatory patients and higher in critically ill and patients which need dialysis [4,5,6,7,8]. In literature reviews, AKI is most commonly reported in surgical and critical settings, where patients are systematically monitored by assessing hourly urinary output and daily creatinine. Despite the lack of extensive data, this syndrome has undeniable importance also in internal medicine wards, where cardiorenal syndrome plays a substantial role [1,2,3,9]. Indeed, AKI occurs in up to 40% of acute decompensated heart failure hospitalizations, which differs according to the criteria used to define AKI [10]. This is known as cardiorenal syndrome type 1 and is an important prognostic factor [10]. Importantly, with the increase in patients with heart failure, the prevalence of this syndrome is also estimated to rise in the near future [9,10]. Mortality rates have declined in critically ill patients, although an increase has been reported in patients with dialysis-requiring AKI [5,6,7,8,9,10,11]. 

AKI is more common in older patients and those with predisposing factors, who present with a higher rate of comorbidities and higher probability of developing severe disease [12]. Sepsis is the leading cause of AKI in critically ill patients, accounting for 50% of cases [13,14]. Furthermore, the differences in patient characteristics, setting, pathophysiology, and outcomes distinguish septic AKI as a separate clinical entity from non-septic AKI [14]. Indeed, septic AKI patients are more likely to require mechanically assisted ventilation and vasoactive drugs, and have longer hospital stays, a higher likelihood of dialysis-requiring AKI, and higher in-hospital mortality rates. Moreover, they have an increased probability of renal function recovery [15,16].

Surgery is another important cause of AKI that accounts for up to 40% of in-hospital AKI cases [17,18]. The highest rates of AKI are found after cardiac (18.7%), general (13.2%), and thoracic (12.0%) surgeries, representing the impact of surgical settings on the incidence variability [19,20].

Recently, the Acute Disease Quality Initiative Workgroup proposed the term acute kidney disease (AKD) to reflect the continuing pathological processes and adverse events developing after AKI [20]. AKD is defined by presenting Kidney Disease Improving Global Outcomes (KDIGO) stage 1 criteria for longer than 7 days after an AKI initiating event [20]. This definition includes the post-AKI period in which critical interventions potentially alter the progression of kidney disease, therefore recognizing a population at risk of chronic kidney disease (CKD) development, cardiovascular events, and mortality [20]. 

Considering the impact of AKI on patient prognosis, research has focused on methods to assess patients at risk for developing AKI and diagnose subclinical AKI, as well as prevention and treatment strategies, for which it is crucial to have an understanding of the epidemiology of AKI. In this review, we discuss the evolving definition and classification of AKI, and its novel diagnostic methods.

## 2. Definitions and Classification

Over the last century, the definition of AKI has evolved significantly [21]. In fact, the diagnosis of AKI has changed from a clinical and biochemical level to a molecular level, with the most recent advances in tubular damage biomarkers increasing the accuracy of the diagnosis [21]. The use of standard classifications to define and stratify AKI has helped to increase the recognition of this disease in clinical practice and epidemiological research, which has led to defining the incidence of AKI in different settings and assessing its association with adverse outcomes [20,21]. This highlighted the importance of prevention, early diagnosis, and prompt treatment of AKI. 

### 2.1. Risk, Injury, Failure, Loss of Kidney Function, End-Stage Kidney Disease (RIFLE) Classification

The RIFLE classification was first published in 2004, resulting from the Acute Dialysis Quality Initiative (ADQI) group conference, which aimed to determine a consensual AKI definition [22]. This classification defines AKI based on variations in serum creatinine (SCr) or estimated glomerular filtration rate (eGFR) and/or urine output (UO), and contemplates three severity levels (risk, injury, and failure) and two outcomes (loss of kidney function and end-stage kidney disease) in AKI [22]. The criteria to use are those that lead to the most negative classification, meaning the maximum RIFLE. The deterioration of renal function from baseline must occur within 7 days and persist for more than 24 h. When baseline SCr is unknown and there is no history of chronic kidney disease, the Modification of Diet in Renal Disease (MDRD) equation should be used to calculate the baseline SCr [23].

The RIFLE classification has been used for determining the incidence of AKI, stratifying AKI severity in multiple settings, and establishing the association between AKI severity and mortality [3,8,24,25]. Despite some limitations, this classification was vital in standardizing the criteria of AKI and confirming AKI severity as an outcome predictor [26].

### 2.2. Acute Kidney Injury Network (AKIN) Classification

In 2007, the AKIN classification was proposed and published by the AKIN working group [27]. There was cumulative evidence demonstrating that small increases in SCr were associated with poor outcomes and that there was variation between hospitals regarding the start of renal replacement therapy, leading to the importance of revising the RIFLE classification [28,29,30].

The AKIN classification depends only on SCr and not on eGFR changes, and does not require baseline SCr, but needs at least two values of SCr obtained within a period of 48 h, thus defining AKI as an increase in SCr of at least 0.3 mg/dL or a percentage increase in SCr equal to or higher than 50%, or by a decrease in UO lower than 0.5 mL/kg/h for more than 6 h. The diagnosis of AKI is only to be considered after achieving an adequate hydration status and excluding urinary obstruction. This classification also excluded the two outcome classes [27].

Both the AKIN and RIFLE classifications led to the identification and stratification of AKI in hospitalized patients, which was independently associated with outcome [31,32,33,34]. The AKIN classification, despite improving diagnostic sensitivity and specificity, shows no evidence of better prognostic acuity [34,35,36,37,38,39].

### 2.3. Kidney Disease Improving Global Outcomes (KDIGO) Classification

Recently, the KDIGO work group has developed a classification by merging the RIFLE and AKIN classifications to provide simplified and integrated criteria that could be applied in clinical practice and research (Table 1) [40]. 

Accordingly, AKI is defined as an increase in SCr of at least 0.3 mg/dL within 48 h, or an increase in SCr to more than 1.5 times of baseline level, which is known or presumed to have occurred within the prior 7 days, or a UO decrease to less than 0.5 mL/kg/h for 6 h. AKI stratification according to KDIGO follows the stages of the AKIN criteria, except for a simplification of stage 3 [40]. 

### 2.4. RIFLE vs. AKIN vs. KDIGO

The KDIGO classification, theoretically, offers superior diagnostic and prognostic accuracy than the former classifications. Recent studies have conducted evaluations of these classifications to assess differences, advantages, and limitations in their incidence determination and prognostic ability in different settings (Table 2).

The Finnaki study demonstrated similar incidence in AKI defined by AKIN and KDIGO in a cohort of 2901 critically patients [41]. Roy et al. also found that the incidence of AKI was similar using the RIFLE, AKIN, and KDIGO criteria in a prospective study of 637 hospitalized patients with acute heart failure, although there were discrete differences in the predictive ability of the 30-day outcomes between RIFLE and KDIGO (area under the receiving operating characteristic curve (AUROC) of 0.76 and 0.74, respectively) [42]. In a retrospective study of 1881 cardiac surgery patients, the RIFLE, AKIN, and KDIGO criteria reported a similar incidence of AKI, although AKIN performed significantly better than RIFLE (AUROC = 0.86 versus 0.78, *p* < 0.001) [43]. Another retrospective cohort study of 31970 hospitalizations reported similar AKI incidence and prognosis using RIFLE, AKIN, and KDIGO [44]. Levi et al. compared the classifications in a study of 190 critical care patients and reported similar incidences [45]. In a prospective study of 1045 hospitalized patients on internal medicine wards conducted by Neves et al., the incidence of AKI was also similar using AKIN and KDIGO criteria, but higher with the RIFLE classification due to the incidence of pre-renal AKI [46].

The KDIGO classification was superior to RIFLE in diagnosing AKI (36.6% versus 14.8%) and predicting early and late mortality (adjusted hazard ratio (HR) for 30-day death of 3.51 by RIFLE and 3.99 by KDIGO; adjusted hazard ratio for 1-year mortality of 1.84 by RIFLE and 2.43 by KDIGO) in a cohort of 1050 patients with acute myocardial infarction [47]. 

The KDIGO criteria demonstrated a higher incidence of AKI than both RIFLE (51% versus 46.9%, *p* = 0.001) and AKIN (51% versus 38.4%, *p* < 0.001) criteria in a prospective cohort of 3107 critically ill patients [47]. Furthermore, evaluating in-hospital mortality, KDIGO was more predictive than RIFLE (*p* < 0.001), but not AKIN (*p* = 0.12) [48]. 

AKI was identified in more patients using the RIFLE and KDIGO criteria than AKIN (11% versus 4.8%) in a retrospective analysis of 49518 hospitalizations [49]. In this study, the KDIGO criteria had superior prognostic ability (AUROC: KDIGO 0.78, RIFLE 0.77, AKIN 0.69) [49]. Li et al. also demonstrated the superior performance of KDIGO in diagnosis and outcome prediction compared to RIFLE and AKIN in a retrospective study of 1005 patients with type 1 cardiorenal syndrome (AUROC: KDIGO 4.00, AKIN 2.68, RIFLE 2.56) [50].

We performed a single-center study of 457 critically ill septic patients and demonstrated that RIFLE and KDIGO criteria identified more AKI cases than did AKIN criteria (RIFLE 84.2% vs. KDIGO 87.5% vs. AKIN 72.8%, *p* < 0.001), although there were no differences in AKI incidence comparing RIFLE and KDIGO classifications, and the prediction of in-hospital mortality was similar between the three classifications [51]. Additionally, in this cohort of septic patients, AKI defined only by UO criteria was a better predictor of in-hospital mortality than was AKI defined either by SCr itself or by both SCr and UO (adjusted odds ratio (OR) = 2.7 (95% CI 1.7–4.5), *p* < 0.001), demonstrating the diagnostic and prognostic importance of UO in patients with septic AKI [51].

In a cohort of 1376 critically ill patients by Koeze et al., the AKIN (15%) and KDIGO (14%) criteria identified more AKI patients than the RIFLE criteria (10%). Moreover, by adding UO criteria, patients were detected earlier than when using only SCr criteria (median time of detection using UO 13 h and SCr 24 h) [52].

The KDIGO classification was also superior to AKIN and RIFLE in predicting in-hospital mortality (AUROC: KDIGO 0.840, AKIN 0.836, RIFLE 0.826, *p* < 0.001) in a study of 167 patients on extracorporeal membrane oxygenation (ECMO) support [53].

Wu et al. performed a retrospective analysis of 826 critically ill surgical patients and demonstrated that KDIGO was a better predictor of in-hospital mortality after surgery than AKIN (AUROC: KDIGO 0.678, AKIN 0.670, *p* <0.001) [54]. 

In a retrospective multi-center cohort of 1036 critically ill patients, the KDIGO criteria identified more AKI patients than RIFLE and AKIN (37.8%, 26.4%, and 34.1%, respectively) [55]. The KDIGO criteria was also a better predictor of mortality (AUROC: KDIGO 0.7013, AKIN 0.6934, RIFLE 0.7016, *p* < 0.001) [55]. Additionally, this study incorporated the Cystatin-C (Cys-C) criteria, which demonstrated good concordance with the RIFLE, AKIN, and KDIGO criteria, and had better predictive ability of mortality than the three definitions (AUROC 0.7023), validating Cys-C as an important biomarker of AKI [55,56].

In a prospective study of 242 critically ill cirrhotic patients, the incidence of AKI was higher with the KDIGO criteria (67%) than with AKIN (65%) or RIFLE (63%), and KDIGO was a better predictor of in-hospital mortality (AUROC: KDIGO 0.781, AKIN 0.741, RIFLE 0.744, *p* < 0.001) [57].

The KDIGO classification appears to perform better in diagnosis and prognosis determination than AKIN and RIFLE. Nonetheless, future prospective studies with larger populations are still required to better assess the sensitivity and prognostic performance of these definitions.

### 2.5. Limitations

Despite the importance of these classifications in defining the epidemiology of AKI, it is increasingly recognized that novel biomarkers have to be researched to improve the definition of AKI and its application in predicting outcomes.

The fact that these classifications rely on SCr, eGFR, and UO, which are insensitive and unspecific markers of AKI and do not account for its duration or cause, is a significant caveat [58]. The value of SCr is influenced by factors altering its production (age, gender, diet, muscle mass), elimination (previous renal dysfunction), secretion (medications) and, importantly, concentration according to fluid balance variations. Baseline SCr is frequently unknown and its assessment is complex, with several studies pointing to the use of minimum preadmission SCr or estimated SCr using the Modification of Diet in Renal Disease formula. Furthermore, UO is difficult to assess without a urinary catheter and can be significantly altered by hypovolemic status and diuretics, and UO adjustment to actual versus ideal body weight affects AKI incidence reports [58,59,60,61,62,63,64].

### 2.6. Future Biomarkers

Recently, potential biomarkers of AKI have been identified. Ideally, novel biomarkers should be specific, identify the cause, identify patients at risk, provide an early diagnosis, stratify the severity of the injury, and predict outcomes. 

With the enhanced understanding of the pathophysiology of AKI, novel biomarkers were identified, including proteins filtered by the glomerulus, enzymes released by tubular cells after injury, and inflammatory mediators [65]. These include Cys-C, neutrophil gelatinase associated lipocalin (NGAL), N-acetyl-glucosaminidase (NAG), kidney injury molecule 1 (KIM-1), interleukin-6 (IL-6), interleukin-8 (IL-8), interleukin 18 (IL-18), liver-type fatty acid-binding protein (L-FABP), calprotectin, urine angiotensinogen (AGT), urine microRNAs, insulin-like growth factor-binding protein 7 (IGFBP7), and tissue inhibitor of metalloproteinases-2 (TIMP-2), which have been evaluated in multiple settings, primarily on critically ill and surgical patients [66,67,68,69,70,71,72,73,74,75,76,77]. 

NGAL was one of the primarily studied biomarkers, which has demonstrated significant prediction of AKI in critically ill, cardiac surgery, sepsis, trauma, and contrast nephropathy patients [65,66,72]. Most recently, the use of IGFBP7 and TIMP-2 has been promising in the critical care setting, demonstrating greater accuracy and stability than former biomarkers [77,78,79,80,81,82]. However, further studies in different clinical settings are still required. 

Most of these biomarkers can be detected in both serum and urine, and have been significantly associated with early AKI prediction. The association of these novel biomarkers with the need for dialysis, renal recovery, progression to CKD, and mortality has also been reported, although further studies are still warranted [65,77].

With recent advances in the understanding of AKI pathogenesis, the role of intrarenal and systemic inflammation leading to multi-organ dysfunction has been emphasized [82,83]. A new marker of systemic inflammation has become available, the neutrophil-lymphocyte ratio (NLR), which has been identified as an AKI prediction tool in multiple settings, being a simple, effective, and low-cost marker [84,85,86].

Despite the current progress in the development of new biomarkers, important drawbacks have limited their widespread applicability in clinical practice. For instance, they have not been able to reliably distinguish pre-renal and renal AKI; moreover, several patient characteristics and comorbidities, such as age, gender, diabetes mellitus, and chronic inflammation, are associated with range variations that limit their validity. The increased cost associated with testing and the need for multiple assessments to increase accuracy limits the cost-effectiveness. Furthermore, evidence of improvement of outcomes associated with using these biomarkers is still lacking [65,77].

Indeed, AKI is a complex syndrome and perhaps the use of a panel of several biomarkers covering different phases of the syndrome could provide a better understanding of its etiology and pathophysiology, and identify targets for future treatments [87].

Additionally, the use of automated electronic alerts (e-alerts) has received much attention in the past few years [88,89]. These consist of algorithms configured from patients’ electronic medical records and clinical information to notify of early or imminent AKI, prompting an earlier clinical evaluation and application of prevention and treatment strategies, potentially improving clinical outcomes [89,90,91,92]. Indeed, a UK consensus conference has encouraged the use of these e-alerts for early detection of AKI [93]. Nevertheless, e-alerts are heterogeneous, do not include clear decision-making strategies, and have not been associated with decreased mortality or renal replacement technique (RRT) use [94]. Further development of these alerts is required to assess their impact on clinical outcomes and recommendation of use in clinical practice. We believe that it is essential to incorporate these scientific advances in daily clinical practice in the near future.

## 3. Conclusions

AKI is a complex syndrome with significant impact on patient outcomes; thus, its prevention, early detection, and prompt treatment are important to minimize the associated morbidity and mortality. 

Research has led to an improvement in our understanding of AKI, raising our awareness of its incidence and prognostic impact. The KDIGO classification unified previous definitions and improved the recognition of AKI in clinical practice. The search for the perfect biomarker of AKI is still ongoing. Future studies should focus on early diagnostic measures, outcome predictors, and new treatments. 

## Figures and Tables

**Table 1:** Risk, Injury, Failure, Loss of kidney function, End-stage kidney disease (RIFLE) [22], Acute Kidney Injury Network (AKIN) [27], and Kidney Disease Improving Global Outcomes (KDIGO) [40] classifications.

Class/Stage	SCr/GFR			UO		
	RIFLE	AKIN	KDIGO	RIFLE	AKIN	KDIGO
Risk/1 *	↑ SCr X 1.5 or ↓ GFR > 25%	↑ SCr ≥ 26.5 μmol/L (≥0.3 mg/dL or ↑ SCr ≥ 150 to 200% (1.5 to 2X)	↑ SCr ≥ 26.5 μmol/L (≥0.3 mg/dL) or ↑ SCr ≥ 150 to 200% (1.5 to 2X)	<0.5 mL/kg/h (>6 h)	<0.5 mL/kg/h (>6 h)	<0.5 mL/kg/h (>6 h)
Injury/2 *	↑ SCr X 2 or ↓ GFR > 50%	↑ SCr > 200 to 300% (>2 to 3X)	↑ SCr > 200 to 300% (>2 to 3X)	<0.5 mL/kg/h (>12 h)	<0.5 mL/kg/h (>12 h)	<0.5 mL/kg/h (>12 h)
Failure/3*	↑ SCr X 3 or ↓ GFR >75% or if baseline SCr ≥ 353.6 μmol/L (≥4 mg/dL) ↑ SCr > 44.2 μmol/L (>0.5 mg/dL)	↑ SCr >300% (>3X) or if baseline SCr ≥ 353.6 μmol/L (≥4 mg/dL) ↑ SCr ≥ 44.2 μmol/L (≥0.5 mg/dL) or initiation of renal replacement therapy	↑ SCr > 300% (>3X) or ↑ SCr to ≥353.6 μmol/L (≥4 mg/dL) or initiation of renal replacement therapy	<0.3 mL/kg/h (>24 h) or anuria (>12 h)	<0.3 mL /kg/h (24 h) or anuria (12 h)	<0.3 mL/kg/h (24 h) or anuria (12 h) or GFR < 35 mL/min/1.73 m^2^ in patients younger than 18 years

SCr: serum creatinine; GFR: glomerular filtration rate; UO: urine output; RIFLE: Risk, Injury, Failure, Loss of kidney function (dialysis dependence for at least 4 weeks), End-stage kidney disease (dialysis dependence for at least 3 months); AKIN: Acute Kidney Injury Network; KDIGO: Kidney Disease Improving Global Outcomes. * Risk class (RIFLE) corresponds to stage 1 (AKIN and KDIGO), Injury class (RIFLE) corresponds to stage 2 (AKIN and KDIGO), and Failure class (RIFLE) corresponds to stage 3 (AKIN and KDIGO), ↑ increase, ↓ decrease.

**Table 2:** Incidence of AKI and patient outcomes according to AKI definitions.

Study	Design	Setting	Criteria	AKI Definition	N	AKI Incidence	Mortality
Nisula et al. (2013) [41]	Prospective, multi-centre	ICU	SCr, UO	AKIN, KDIGO	2901	AKIN 39.3% KDIGO 39.3%	AKIN 26% KDIGO 26%
Roy et al. (2013) [42]	Prospective	Hospitalized, HF	SCr	RIFLE, AKIN, KDIGO	637	RIFLE 25.6%, AKIN 27.9%, KDIGO 36.7%	RIFLE AUROC 0.76 AKIN AUROC 0.72 KDIGO AUROC 0.74 *p* = 0.02
Bastin et al. (2013) [43]	Retrospective	Cardiac surgery	SCr	RIFLE, AKIN, KDIGO	1881	RIFLE 24.9%, AKIN 25.9%, KDIGO 25.9%	RIFLE AUROC 0.78, AKIN AUROC 0.86, *p* < 0.001
Zeng et al. (2014) [44]	Retrospective	Hospitalized	SCr	RIFLE, AKIN, KDIGO	31,970	RIFLE 16.1%, AKIN 16.6%, KDIGO 18.3%	RIFLE OR 2.9, AKIN OR 2.6, KDIGO OR 2.8
Levi et al. (2013) [45]	Prospective	ICU	SCr, UO	RIFLE, AKIN, KDIGO	190	RIFLE 62.6%, AKIN 63.2%, KDIGO 63.2%	RIFLE OR 0.56, AKIN OR 0.58, KDIGO OR 0.58
Rodrigues et al. (2013) [46]	Prospective	AMI	SCr	RIFLE, KDIGO	1050	RIFLE 14.8% KDIGO 36.6%	RIFLE HR 3.51 (early) 1.84 (late) KDIGO HR 3.99 (early) 2.43 (late)
Luo et al. (2014) [47]	Prospective	ICU	SCr, UO	RIFLE, AKIN, KDIGO	3107	RIFLE 46.9%, AKIN 38.4%, KDIGO 51% *p* = 0.001	RIFLE AUROC 0.738 AKIN AUROC 0.746 KDIGO AUROC 0.757 KDIGO vs. RIFLE *p* = 0.12 KDIGO vs. AKIN *p* < 0.001
Fuji et al. (2014) [48]	Retrospective	Hospitalized	SCr	RIFLE, AKIN, KDIGO	49,518	RIFLE 11.0%, AKIN 4.8%, KDIGO 11.8%	RIFLE AUROC 0.77 AKIN AUROC 0.69 KDIGO AUROC 0.78 *p* = 0.02
Neves et al. (2014) [49]	Prospective	Hospitalized	SCr, UO	RIFLE, AKIN, KDIGO	1045	RIFLE 6.2%, AKIN 5.5%, KDIGO 5.5%	N/A
Li et al. (2014) [50]	Retrospective	Hospitalized	SCr	RIFLE, AKIN, KDIGO	1005	RIFLE 32.1%, AKIN 34.7%, KDIGO 38.9%	RIFLE OR 2.56 AKIN OR 2.68 KDIGO OR 4.00 *p* < 0.05
Pereira et al. (2017) [51]	Retrospective	ICU, Sepsis	SCr, UO	RIFLE, AKIN, KDIGO	457	RIFLE 84.2%, AKIN 72.8%, KDIGO 87.5%	RIFLE AUROC 0.652 AKIN AUROC 0.686 KDIGO AUROC 0.658 *p* < 0.001
Koeze et al. (2017) [52]	Retrospective	ICU	SCr, UO	RIFLE, AKIN, KDIGO	1376	RIFLE 28% (SCr) 35% (SCr + UO) AKIN 12% (SCr) 38% (SCr + UO) KDIGO 11% (SCr) 38% (SCr + UO)	RIFLE 84.2%, AKIN 72.8%, KDIGO 87.5%
Tsai et al. (2017) [53]	Retrospective	ECMO	SCr, UO	RIFLE, AKIN, KDIGO	167	RIFLE 75.4%, AKIN 84.4%, KDIGO 85%	RIFLE AUROC 0.826 AKIN AUROC 0.774 KDIGO AUROC 0.840 *p* < 0.001
Wu et al. (2016) [54]	Retrospective	ICU, Surgical	SCr, UO	AKIN, KDIGO	826	AKIN 31% KDIGO 30%	AKIN 21.8% (1), 20.2% (2), 27.8% (3) KDIGO 16.9% (1), 17.5% (2), 34.1% (3)
Zhou et al. (2016) [55]	Retrospective	ICU	SCr, UO, Cys-C	RIFLE, AKIN, KDIGO	1036	RIFLE 26.4%, AKIN 34.1%, KDIGO 37.8%, Cys-C 36.1%	RIFLE 57.9%, AKIN 54.4%, KDIGO 51.8%, Cys-C 52.1%
Pan et al. (2016) [56]	Retrospective	ICU, Cirrhosis	SCr, UO	RIFLE, AKIN, KDIGO	242	RIFLE, AKIN, KDIGO	RIFLE AUROC 0.774 AKIN AUROC 0.741 KDIGO AUROC 0.781 *p* < 0.001

ICU: Intensive care unit, SCr: Serum creatinine, UO: Urinary output, HF: Heart failure, AMI: acute myocardial infarction, Cys-C: Cystatin C, N/A not applicable, AUROC: area under the receiving operating characteristic curve, HR: hazard ratio, OR: odds ratio.
